# Supplementary figures and images for: The RBR E3 ubiquitin ligase HOIL-1 can ubiquitinate diverse non-protein substrates in vitro
Source: Life Sci Alliance. 2025 Apr 1;8(6):e202503243. doi: 10.26508/lsa.202503243 (PMC11962058; doi:10.26508/lsa.202503243)

Source data for Figure 1

**A**

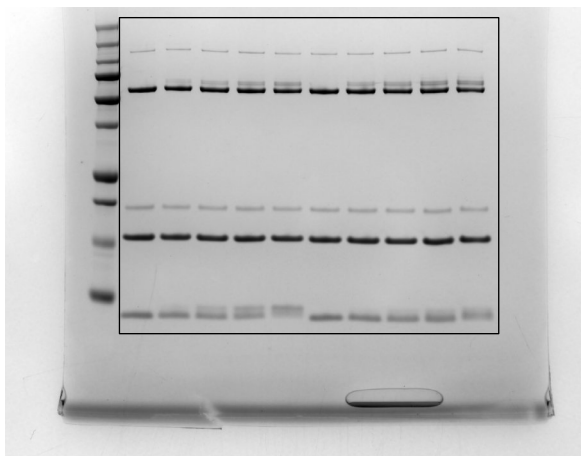

**B**

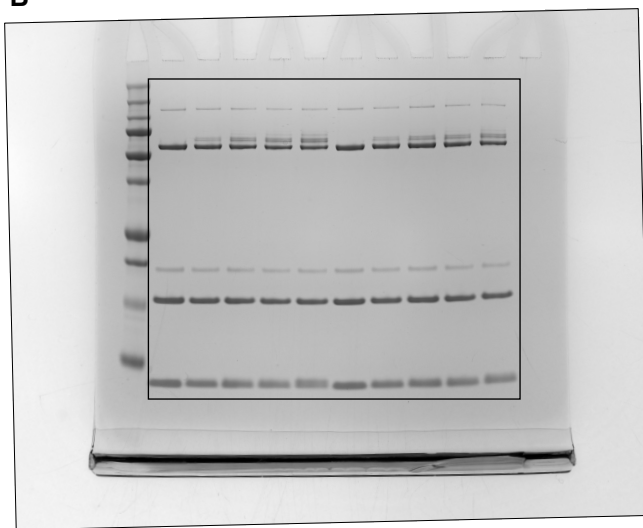

**C**

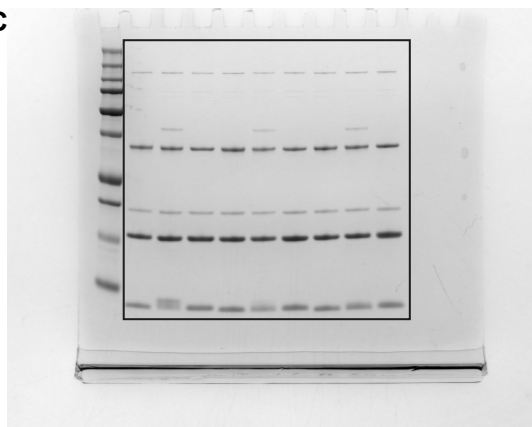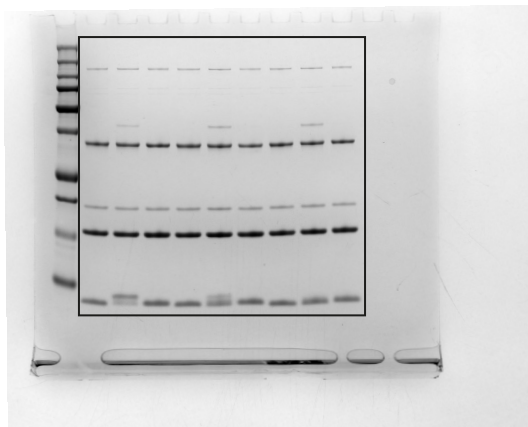

**D**

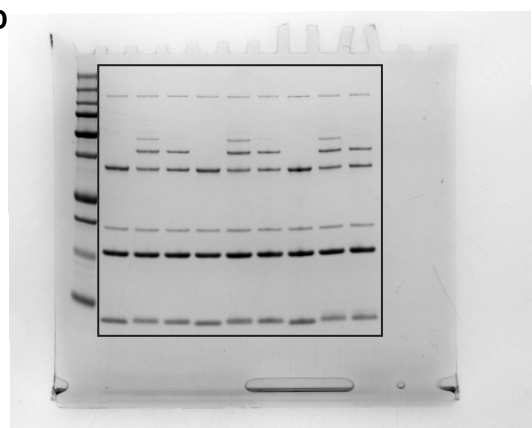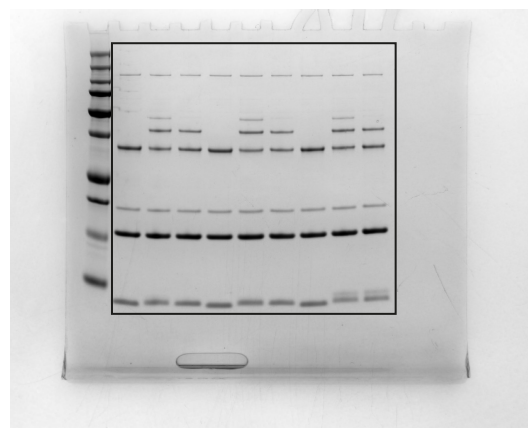

Supplement: Supplementary file 1 [file LSA-2025-03243_SdataF1.pdf]

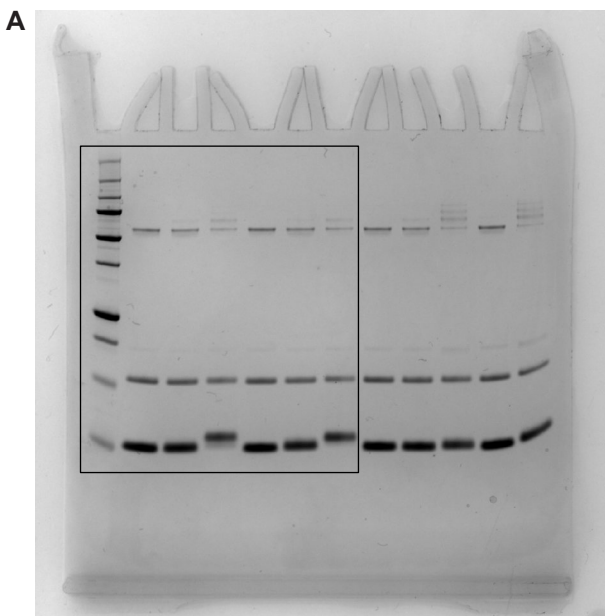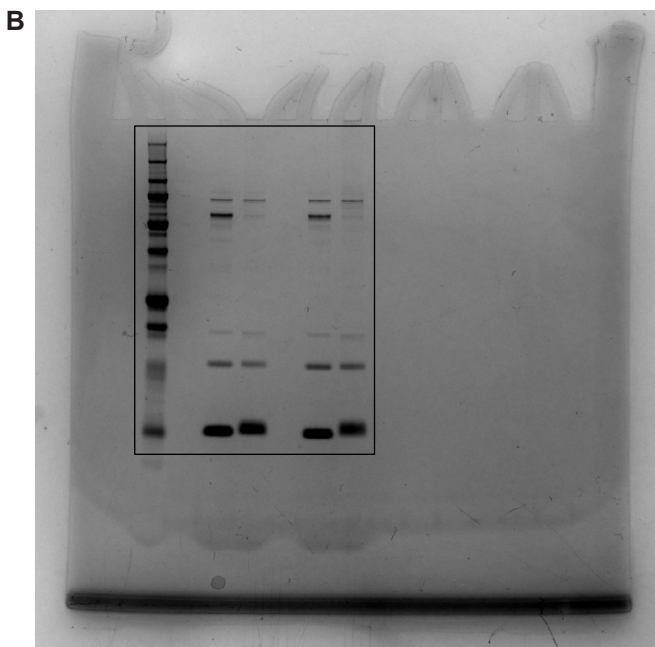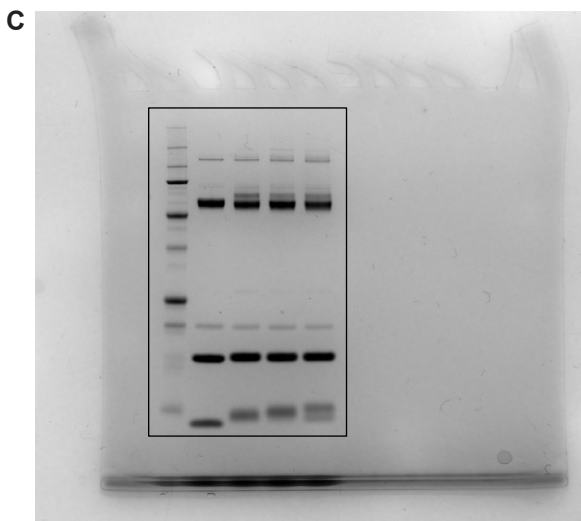

Supplement: Supplementary file 5 [file LSA-2025-03243_SdataFS3.pdf]

**B**

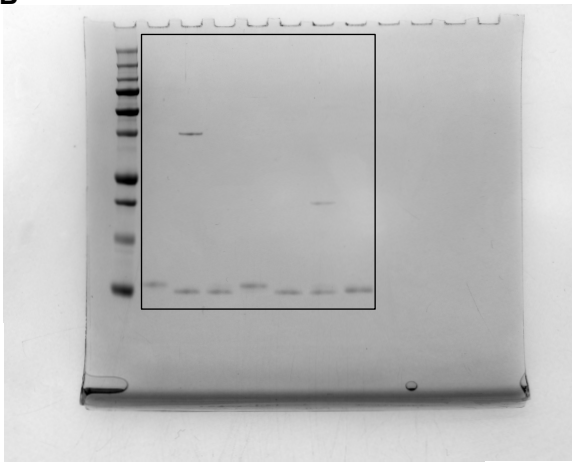

Supplement: Supplementary file 7 [file LSA-2025-03243_SdataF5.pdf]

Source data for Figure S5

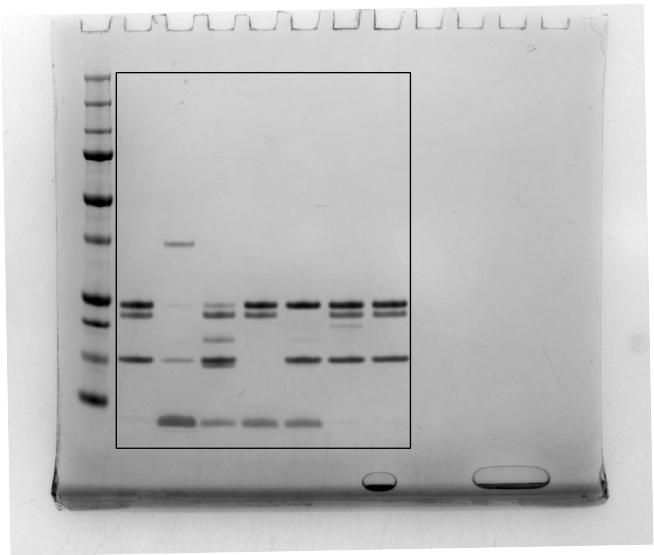

Supplement: Supplementary file 8 [file LSA-2025-03243_SdataFS5.pdf]

Source data for Figure 6

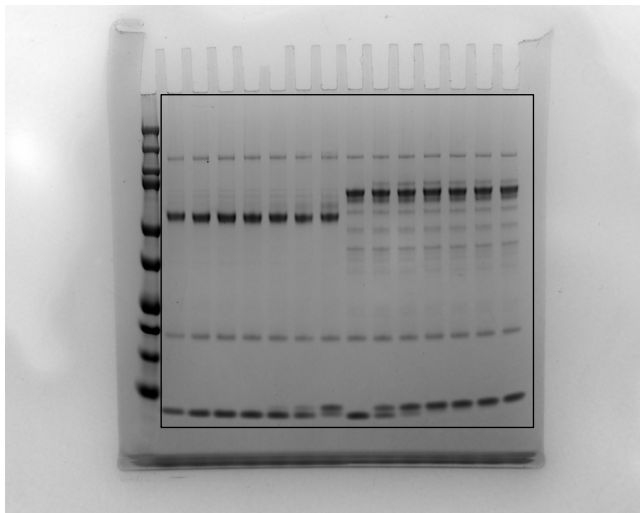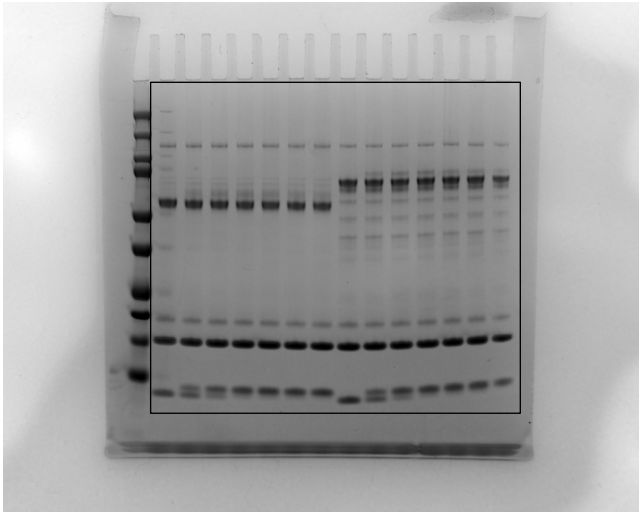

Supplement: Supplementary file 9 [file LSA-2025-03243_SdataF6.pdf]

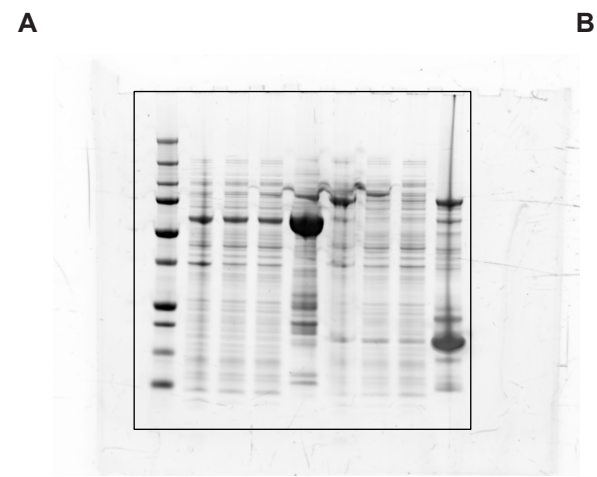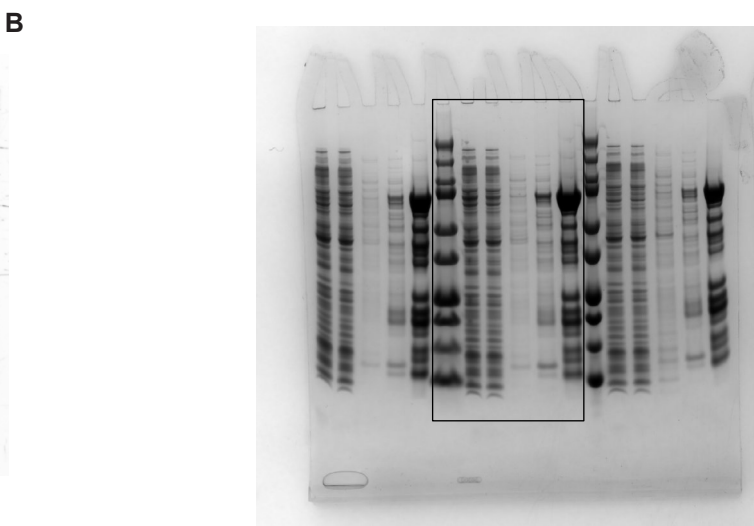

Supplement: Supplementary file 10 [file LSA-2025-03243_SdataFS6.pdf]
